# Proteomic Analysis of Tear Film in Dogs and Cats: Emerging Biomarkers of Cognitive Dysfunction and Neurodegenerative Disorders

**DOI:** 10.3390/ani16060930

**Published:** 2026-03-16

**Authors:** Dagmara Winiarczyk, Mateusz Winiarczyk

**Affiliations:** 1 Department of Internal Diseases of Small Animals, University of Life Sciences of Lublin, 20-950 Lublin, Poland; 2 Department of Vitreoretinal Surgery, Medical University of Lublin, 20-950 Lublin, Poland; mateuszwiniarczyk@umlub.pl

**Keywords:** cognitive dysfunction, aging, tear film, proteomic analysis, biomarkers

## Abstract

Cognitive dysfunction and neurodegenerative disorders represent an increasing clinical challenge in aging dogs and cats, while objective and minimally invasive biomarkers for early detection and disease monitoring remain limited. Tear film is a biologically active fluid reflecting both local and systemic processes and offers a practical, non-invasive source of potential biomarkers in geriatric veterinary patients. Proteomic analyses of canine and feline tear film have revealed a complex protein composition, including molecules involved in inflammation, oxidative stress, immune regulation, and cellular homeostasis—processes implicated in neurodegeneration. However, growing evidence from human and veterinary research emphasizes the importance of CNS-specific and mechanistically informative biomarkers, such as markers of axonal injury, synaptic degeneration, and glial activation, which may provide a more precise framework for interpreting peripheral proteomic alterations. This review summarizes current knowledge on tear film proteomics in dogs and cats and discusses its potential relevance to cognitive dysfunction and neurodegenerative processes. Particular attention is given to the integration of tear-derived proteins with validated blood and cerebrospinal fluid biomarkers, as well as to methodological challenges and future research priorities. With appropriate standardization and clinical validation, tear film proteomics may contribute to the development of novel diagnostic and monitoring strategies for neurodegenerative disorders in companion animals.

## Simple Summary

Cognitive dysfunction and neurodegenerative disorders are increasingly recognized in aging dogs and cats and represent an important and growing challenge in veterinary practice. These conditions are commonly diagnosed based on behavioral changes reported by owners, while objective and easily accessible biological markers that could support early detection or disease monitoring remain limited. Therefore, there is an important need for non-invasive diagnostic tools that can be applied safely and repeatedly in geriatric companion animals. Tear film, a fluid covering the ocular surface, contains numerous proteins derived from local tissues as well as from systemic circulation. In recent years, advances in proteomic techniques have enabled detailed analysis of the tear film protein composition in dogs and cats. Many of the identified proteins are involved in inflammation, oxidative stress, immune regulation, and cellular maintenance, which are biological processes known to play an important role in neurodegenerative changes and age-related cognitive decline in animals. These findings suggest that tear-derived proteins may provide indirect information on molecular alterations associated with cognitive dysfunction. This review summarizes current knowledge on proteomic studies of tear film in dogs and cats and discusses the potential relevance of tear-derived proteins as biomarkers of cognitive dysfunction and neurodegenerative processes. The advantages of tear film as a non-invasive and clinically practical sample are highlighted, along with current limitations of available studies. Improved understanding of tear film proteomics may contribute to the development of novel diagnostic and monitoring tools for cognitive dysfunction in companion animals.

## 1. Introduction

Cognitive dysfunction and neurodegenerative disorders are increasingly recognized in aging dogs and cats, reflecting the growing lifespan of companion animals and improved awareness among veterinarians and owners [1,2]. Cognitive dysfunction syndrome (CDS) in dogs and analogous age-related cognitive impairment described in cats are characterized by progressive behavioral changes, including disorientation, altered social interactions, sleep–wake cycle disturbances, and changes in activity levels [1,3,4,5]. These clinical signs significantly affect animal welfare and owner–animal relationships; however, diagnosis remains challenging, particularly in early disease stages.

At present, the diagnosis of cognitive dysfunction in companion animals relies largely on behavioral assessment tools and owner-based questionnaires, often supported by the exclusion of other systemic or neurological conditions [1,2,6,7]. Objective biological markers that could aid in early diagnosis, disease staging, or monitoring of disease progression are still lacking in routine veterinary practice. This limitation highlights an important need for accessible, reliable, and minimally invasive biomarkers that can be applied repeatedly, especially in geriatric patients.

Neurodegenerative processes associated with cognitive dysfunction in dogs and cats involve complex pathophysiological mechanisms, including chronic neuroinflammation, oxidative stress, altered protein homeostasis, and impaired cellular maintenance within the central nervous system [2,8]. These mechanisms are not restricted to neural tissue and may be reflected in peripheral biological fluids. Consequently, increasing attention has been directed toward identifying biomarkers in easily obtainable samples such as blood, urine, saliva, and ocular secretions [2,8]. These findings suggest that peripheral matrices may provide indirect information on ongoing neurodegenerative processes.

Tear film is a complex biological fluid composed of proteins, lipids, metabolites, and electrolytes originating from the lacrimal glands, ocular surface tissues, and systemic circulation [9,10]. In veterinary medicine, tear film analysis has been analyzed and recent advances in proteomic technologies have revealed that tear film composition may reflect systemic and neurological processes [10,11,12,13,14,15]. Importantly, tear collection is non-invasive, well tolerated, and suitable for repeated sampling, which is particularly important for use in aging dogs and cats.

Mass spectrometry-based proteomic approaches have enabled detailed characterization of the canine and feline tear film proteome, identifying proteins involved in immune response, inflammation, oxidative stress regulation, and cellular homeostasis [10,11,16]. Many of these biological pathways are implicated in neurodegenerative changes and age-related cognitive decline in companion animals [2,8,17]. Despite this potential, the relevance of tear film proteomics to cognitive dysfunction and neurodegenerative disorders in dogs and cats remains insufficiently explored, and available data are still limited.

The aim of this review is to summarize current knowledge on proteomic analyses of tear film in dogs and cats, with a focus on proteins potentially associated with cognitive dysfunction and neurodegenerative processes. By discussing methodological approaches, biological relevance, and clinical implications, this review highlights the potential of tear film proteomics as a novel, non-invasive tool for advancing the understanding, diagnosis, and monitoring of cognitive dysfunction in companion animals.

## 2. Tear Film as a Source of Biomarkers in Dogs and Cats

Tear film is a complex and dynamic biological fluid that plays an important role in maintaining ocular surface homeostasis in dogs and cats [18]. It is composed of three interactive layers—lipid, aqueous, and mucin—produced by the meibomian glands, lacrimal glands, and conjunctival goblet cells, respectively [18]. Beyond its protective and lubricating functions, tear film contains a wide range of biologically active molecules, including proteins, lipids, metabolites, electrolytes, and signaling molecules, which may reflect both local ocular conditions and systemic physiological processes [9,11].

Proteins present in tear film originate from multiple sources, including secretion by lacrimal and accessory glands, diffusion from plasma, and local synthesis by ocular surface epithelial and immune cells [9,11]. As a result, the tear film proteome encompasses proteins involved in immune defense, inflammation, oxidative stress regulation, cellular maintenance, and tissue remodeling [10,11,16]. In veterinary medicine, these characteristics have primarily been explored in the context of ocular surface diseases; however, increasing evidence suggests that tear film composition may also mirror broader systemic alterations [10,11].

Compared with other biological fluids commonly used for biomarker discovery, such as blood or cerebrospinal fluid, tear film offers several practical and clinically important advantages. Tear collection is minimally invasive, does not require sedation or anesthesia, and is well tolerated by dogs and cats, including geriatric and neurologically compromised patients [18,19]. Furthermore, tear sampling allows for repeated measurements over time, facilitating longitudinal monitoring of disease progression or therapeutic response, which is particularly important in chronic conditions.

In recent years, advances in analytical techniques have enabled more comprehensive characterization of the canine and feline tear film proteome. Proteomic studies have identified numerous proteins associated with inflammatory pathways, oxidative stress responses, immune regulation, and cellular homeostasis, which are biological processes known to be involved in age-related cognitive decline and neurodegenerative changes in companion animals [10,11,12,14,16,20,21]. Importantly, some tear film proteins have been shown to correlate with systemic conditions, supporting the concept that tear fluid may serve as a surrogate matrix for biomarker discovery beyond ophthalmology. These findings suggest that tear film proteomics may provide indirect information on molecular alterations occurring in different disease states.

Despite these promising features, the use of tear film as a source of biomarkers in veterinary neurology remains largely unexplored. Variability related to tear collection methods, environmental factors, breed differences, and age must be carefully considered when interpreting tear proteomic data [10,12,15]. It is important to note that ocular surface integrity and concurrent ophthalmic diseases may also influence tear composition and represent potential confounding factors [22]. Nevertheless, the unique accessibility and biological richness of tear film position it as an important and underutilized fluid for the identification of biomarkers relevant to cognitive dysfunction and neurodegenerative disorders in dogs and cats.

## 3. Proteomic Methodologies Applied to Tear Film Analysis

Proteomic analysis of tear film in dogs and cats requires carefully optimized methodologies due to the small sample volume, complex protein composition, and susceptibility of tears to environmental and technical variability. Over the past two decades, methodological advances in sample collection, preparation, and mass spectrometry-based analysis have enabled increasingly detailed characterization of the canine and feline tear film proteome. Standardization of these procedures is important to ensure reproducibility and reliable interpretation of results, particularly when tear-derived proteins are considered as potential biomarkers of cognitive dysfunction and neurodegenerative disorders in companion animals.

### 3.1. Tear Collection Methods and Pre-Analytical Considerations

The method of tear collection represents a critical pre-analytical factor influencing both the qualitative and quantitative composition of the tear proteome. In dogs and cats, tears are most commonly collected using Schirmer tear test (STT) strips or microcapillary tubes. STT strips are widely available, inexpensive, and easy to use in clinical practice; however, they may induce reflex tearing and mechanical stimulation of the ocular surface, potentially altering protein composition and diluting basal tear components [19,22,23,24]. In contrast, microcapillary tubes allow collection of basal tears with minimal stimulation but require greater technical expertise and patient cooperation [19].

Additional pre-analytical variables include the duration of collection, time of day, environmental conditions, recent ophthalmic manipulation, and the presence of ocular or systemic disease. Breed, age, and sex have also been shown to influence tear protein profiles in dogs [25,26]. Following collection, prompt and standardized processing is important. Tear samples are typically eluted from collection devices using buffered solutions, centrifuged to remove debris, and stored at −80 °C to preserve protein integrity prior to proteomic analysis.

### 3.2. Sample Preparation and Protein Extraction

Due to the limited volume of tear fluid obtainable from companion animals, efficient protein extraction and concentration strategies are required. Protein recovery from STT strips or capillary samples is commonly achieved using phosphate-buffered saline or ammonium bicarbonate-based buffers, followed by centrifugation and filtration. Protein concentration may be enhanced using ultrafiltration devices or precipitation techniques [27,28,29].

Prior to mass spectrometric analysis, tear proteins are usually subjected to reduction, alkylation, and enzymatic digestion, most commonly with trypsin. Given the dynamic range of tear proteins, depletion of highly abundant proteins or fractionation strategies may be applied to improve detection of low-abundance components with potential biomarker relevance. However, it is important to note that such procedures may introduce bias and must be carefully validated.

### 3.3. Mass Spectrometry-Based Proteomic Approaches

Modern tear film proteomics in veterinary medicine relies primarily on liquid chromatography coupled with tandem mass spectrometry (LC–MS/MS). Early studies employed gel-based separation techniques combined with matrix-assisted laser desorption/ionization (MALDI) or electrospray ionization mass spectrometry, whereas current approaches increasingly utilize high-resolution, label-free quantitative LC–MS/MS platforms [20,28].

Shotgun proteomics enables unbiased identification of hundreds to thousands of proteins from small tear volumes and has been successfully applied to characterize the tear proteome in healthy dogs and cats as well as in animals with ocular disease [15]. Quantitative strategies, including label-free spectral counting, intensity-based quantification, and data-independent acquisition (DIA), allow comparison of protein abundance between experimental groups, such as young versus geriatric animals or cognitively normal versus cognitively impaired patients.

Bioinformatic analysis constitutes an integral component of tear proteomic workflows. Identified proteins are commonly subjected to functional annotation and pathway analysis to explore biological processes related to inflammation, oxidative stress, immune regulation, and cellular maintenance. These analyses are particularly important when investigating neurodegenerative mechanisms associated with age-related cognitive decline in companion animals.

### 3.4. Technical Challenges and Sources of Variability

Despite substantial methodological progress, tear film proteomics remains technically challenging. The small sample volume, high inter-individual variability, and influence of environmental and physiological factors complicate standardization and data interpretation. Differences in collection devices, elution protocols, digestion methods, and mass spectrometric platforms may result in inconsistent protein identification across studies [27].

In addition, the absence of species-specific protein databases for certain companion animals and incomplete annotation of canine and feline proteomes may limit accurate protein identification and functional interpretation. These limitations underscore the importance of rigorous experimental design, inclusion of appropriate control groups, and transparent reporting of methodological details in future studies.

### 3.5. Relevance of Proteomic Methodologies to Biomarker Discovery in Cognitive Dysfunction

Proteomic methodologies provide a powerful and unbiased approach for identifying tear-derived proteins potentially associated with neurodegenerative processes and cognitive dysfunction in dogs and cats. Importantly, the non-invasive nature of tear collection facilitates longitudinal sampling, making proteomic approaches particularly suitable for monitoring disease progression and therapeutic responses in geriatric patients.

Continued refinement and standardization of tear proteomic workflows will be essential to translate experimental findings into clinically applicable biomarkers. Integration of proteomic data with behavioral assessment, imaging findings, and other biological matrices may further enhance the diagnostic and prognostic value of tear-derived biomarkers in veterinary neurology.

## 4. Tear Film Proteins Associated with Neurodegenerative Processes

Neurodegenerative disorders and age-related cognitive dysfunction in dogs and cats are characterized by complex molecular alterations involving neuroinflammation, oxidative stress, impaired protein homeostasis, immune dysregulation, and progressive cellular dysfunction within the central nervous system. Although these processes primarily affect neural tissue, increasing evidence indicates that systemic and peripheral biological fluids may reflect ongoing neurodegenerative changes. Due to its diverse protein composition and close relationship with systemic circulation, tear film represents a promising matrix for investigating molecular pathways associated with neurodegeneration in companion animals.

Proteomic studies of canine and feline tear film have identified a broad spectrum of proteins involved in biological processes that are known to contribute to cognitive decline and neurodegenerative pathology [30,31,32]. Importantly, recent serum proteomic studies in dogs have demonstrated that cognitive dysfunction is associated with measurable systemic alterations, including differential abundance of specific proteins linked to inflammation, oxidative stress, and neurodegenerative pathways [31]. These observations support the concept that peripheral biofluids may reflect central nervous system pathology. Therefore, it is plausible that tear film proteomics could also provide clinically relevant information and contribute to the identification of non-invasive biomarkers for canine cognitive dysfunction.

### 4.1. Proteins Associated with Neuroinflammation and Immune Regulation

Chronic, low-grade neuroinflammation is considered an important mechanism underlying age-related cognitive decline in companion animals. Inflammatory mediators produced within the central nervous system may influence peripheral immune responses and contribute to altered protein profiles in biological fluids, including tear film. Proteomic analyses of canine tear film have identified several proteins involved in innate and adaptive immune regulation, such as complement components, acute-phase proteins, and immunoglobulin-related proteins [12,21].

Proteins belonging to the S100 family, complement factors, and other inflammation-related molecules have been consistently detected in canine tear samples and are known to participate in leukocyte activation, cytokine signaling, and tissue remodeling. Alterations in the abundance of such proteins may reflect systemic inflammatory status associated with neurodegenerative processes. It is important to note that, given the recognized role of neuroinflammation in cognitive dysfunction in dogs and cats, tear-derived inflammatory proteins represent promising candidates for further investigation as peripheral indicators of central nervous system pathology.

### 4.2. Oxidative Stress-Related Proteins

Oxidative stress is a key contributor to neuronal damage and synaptic dysfunction during aging and neurodegeneration. An imbalance between reactive oxygen species production and antioxidant defense mechanisms leads to cumulative cellular injury and impaired neuronal function. In tear film proteomes of dogs and cats, multiple proteins involved in antioxidant defense and redox regulation have been identified, including peroxiredoxins, superoxide dismutase, glutathione-related enzymes, and heat shock proteins [13,21,33].

These proteins contribute to cellular redox homeostasis and protection against oxidative damage. Alterations in their abundance or post-translational modification may reflect increased oxidative burden associated with age-related cognitive decline, suggesting that tear-derived antioxidant proteins could serve as peripheral indicators of oxidative stress in cognitive dysfunction.

### 4.3. Proteins Involved in Cellular Homeostasis and Protein Quality Control

Maintenance of protein homeostasis is essential for neuronal survival and synaptic integrity. Disruption of protein folding, degradation, and intracellular trafficking contributes to the accumulation of damaged proteins and progressive cellular dysfunction in neurodegenerative disorders [31]. Tear proteomic studies have detected several chaperones and proteostasis-related proteins, including heat shock proteins, ubiquitin-related enzymes, and cytoskeletal components [34,35].

Heat shock proteins act as molecular chaperones that stabilize unfolded proteins and facilitate their degradation, thereby limiting proteotoxic stress. Alterations in the abundance of these proteins may indicate impaired cellular stress responses associated with neurodegeneration. Similarly, changes in cytoskeletal and vesicle-associated proteins detected in tear film may reflect disturbances in intracellular transport and synaptic maintenance occurring during cognitive decline.

### 4.4. Proteins Related to Neuronal Structure and Synaptic Function

Although tear film does not originate directly from neural tissue, several proteins associated with neuronal maintenance and cytoskeletal organization have been identified in canine tear proteomes. These include actin-binding proteins, intermediate filament components, and vesicle trafficking proteins that are essential for synaptic stability and neurotransmission [13,25].

Age-related alterations in synaptic proteins and cytoskeletal dynamics are known to contribute to impaired neuronal connectivity and cognitive performance in dogs and cats. Detection of such proteins in tear film supports the concept that peripheral fluids may partially reflect central neurodegenerative processes. It is important to note that further studies are required to determine whether quantitative changes in these proteins correlate with cognitive status or disease progression in companion animals.

### 4.5. Perspectives on Candidate Tear Film Biomarkers of Neurodegeneration

Taken together, the functional classes of proteins identified in canine and feline tear film overlap substantially with molecular pathways implicated in neurodegenerative disorders and cognitive dysfunction. Although current evidence remains largely indirect, these findings highlight the potential of tear film proteomics to provide novel insights into peripheral manifestations of central nervous system pathology. Table 1 provides a simplified overview of tear-derived protein categories discussed in Section 4 and their potential relevance to biological processes implicated in neurodegeneration.

## 5. Tear Film Biomarkers and Cognitive Dysfunction in Dogs and Cats

Objective assessment of cognitive dysfunction in companion animals remains an important challenge in veterinary neurology. Current diagnosis relies primarily on behavioral evaluation and owner-based questionnaires, which are inherently subjective and often insensitive to early or subtle disease stages. Consequently, there is an important and growing interest in identifying peripheral biomarkers that could support early detection, improve diagnostic accuracy, and allow monitoring of disease progression and therapeutic response in dogs and cats with cognitive dysfunction.

Tear film represents a particularly attractive source of biomarkers for this purpose because of its non-invasive collection, suitability for repeated sampling, and rich protein composition reflecting both local and systemic processes. Although direct studies evaluating tear-derived biomarkers specifically in canine or feline cognitive dysfunction are currently scarce, accumulating evidence from proteomic analyses of tear film and from studies of systemic biomarkers in aging dogs supports the feasibility of this approach. These findings suggest that tear film proteomics may provide indirect information on molecular alterations associated with cognitive dysfunction.

### 5.1. Current Biomarker Strategies in Canine and Feline Cognitive Dysfunction

To date, most biomarker research in cognitive dysfunction of companion animals has focused on blood- and cerebrospinal fluid-derived parameters, including markers of inflammation, oxidative stress, and neuronal injury. Several studies have reported associations between circulating inflammatory mediators, such as C-reactive protein and interleukin-6, and cognitive performance in aging dogs, supporting a role for systemic inflammation in cognitive decline [36]. Likewise, increased levels of neurodegeneration-associated proteins such as neurofilament light chain (NfL) have been detected in both plasma and cerebrospinal fluid of dogs with presumptive cognitive dysfunction, indicating correlations between peripheral biomarkers and neuropathology [37]. However, the invasive nature of cerebrospinal fluid sampling and the variability of serum biomarkers limit their routine clinical applicability.

### 5.2. CNS-Specific Biomarkers of Neurodegeneration: Mechanistic and Translational Relevance

Increasing attention has been directed toward biomarkers that directly reflect CNS-specific neurodegenerative mechanisms rather than systemic inflammatory or oxidative processes. Among these, markers of axonal injury, synaptic degeneration, astroglial activation, microglial activation, and proteostatic dysfunction have demonstrated strong mechanistic and diagnostic value in human neurodegenerative diseases and are increasingly explored in canine cognitive dysfunction (CCD).

#### 5.2.1. Axonal Injury

Neurofilament light chain (NfL) is currently the most robust and widely validated fluid biomarker of neuroaxonal injury. Elevated NfL concentrations in CSF and blood correlate with disease severity and progression in Alzheimer’s disease (AD), frontotemporal dementia, and other neurodegenerative disorders [38,39,40]. Plasma NfL levels rise early in disease and reflect ongoing neurodegeneration [40].

In veterinary medicine, plasma and CSF NfL have been proposed as translational biomarkers in aging dogs and CCD [41,42]. Elevated circulating NfL concentrations have been observed in dogs with cognitive decline and other CNS disorders, supporting its potential as a minimally invasive biomarker [41]. Among currently available candidates, NfL represents the most promising bridge between human and canine neurodegenerative research.

#### 5.2.2. Synaptic Degeneration

Synaptic loss is a major correlate of cognitive impairment. In human AD, CSF neurogranin and SNAP-25 reflect synaptic dysfunction and predict disease progression [43]. These biomarkers provide mechanistic insight beyond general inflammatory processes.

Although synaptic protein biomarkers have not yet been validated in canine tear fluid, histopathological and molecular evidence indicates synaptic alterations in aging dogs with cognitive impairment [44]. Exploration of synaptic proteins in tear proteomics may therefore represent a mechanistically relevant translational avenue.

#### 5.2.3. Astrocytic Activation

Glial fibrillary acidic protein (GFAP) is an established marker of astrocyte activation and astroglial injury. Plasma GFAP increases early in AD and correlates with amyloid pathology [45,46]. Importantly, plasma GFAP may rise even before overt neurodegeneration becomes clinically apparent [45].

Astroglial activation has been documented in canine neurodegenerative conditions, suggesting translational potential for astrocyte-related biomarkers in veterinary medicine [44]. Compared to nonspecific inflammatory markers, GFAP provides improved CNS specificity.

#### 5.2.4. Microglial Activation

Microglial activation is central to neurodegenerative progression. Soluble TREM2 (sTREM2) and YKL-40 (CHI3L1) are recognized markers of microglial activation in human AD and correlate with disease progression [47]. These biomarkers reflect innate immune activation within the CNS rather than systemic inflammation.

While veterinary validation remains limited, microglial activation is a well-described feature of CCD neuropathology [44]. The identification of microglia-associated proteins in tear film could provide novel mechanistic insight.

#### 5.2.5. Proteostasis and Lysosomal Dysfunction

Impaired autophagy and lysosomal pathways contribute to protein aggregation and neuronal loss in neurodegenerative diseases. Dysregulation of cathepsins and lysosomal-associated membrane proteins has been implicated in AD pathogenesis [48]. Given the central role of proteostasis in both human AD and CCD, biomarkers reflecting lysosomal/autophagic dysfunction warrant investigation in tear proteomics.

#### 5.2.6. Current Status of Blood and CSF Biomarkers in Dogs and Cats

Among fluid biomarkers investigated in dogs, NfL currently demonstrates the strongest translational evidence [41,42]. Amyloid-β peptides have been detected in plasma and CSF of aged dogs with cognitive dysfunction, though variability remains high [49,50]. GFAP and tau have primarily been studied in acute neurological conditions, and their role in naturally occurring CCD requires further investigation.

In cats, data on validated blood or CSF biomarkers of neurodegenerative disease remain extremely limited, highlighting a significant translational research gap.

Overall, the integration of CNS-specific biomarkers—particularly those reflecting axonal injury, synaptic degeneration, astrocytic activation, and proteostatic dysfunction—may substantially enhance the specificity and translational relevance of tear film proteomics in veterinary neurodegeneration.

A structured overview of the most relevant CNS-specific biomarkers, categorized according to pathophysiological processes and translational relevance, is presented in Table 2.

### 5.3. Tear Film Biomarkers in Veterinary Neurodegeneration: From Systemic Signals to CNS-Specific Targets

Tear fluid represents a biologically accessible and minimally invasive matrix that reflects both systemic and potentially neuro-related molecular changes. While early tear proteomics studies primarily identified markers associated with oxidative stress, inflammation, and immune modulation, increasing attention is being directed toward the detection of proteins that may more directly reflect CNS-specific neurodegenerative mechanisms [53].

#### 5.3.1. Systemic and Neuroimmune Signals in Tear Fluid

Tear-derived inflammatory and oxidative stress-related proteins may reflect systemic manifestations accompanying neurodegenerative processes. However, although biologically relevant, such markers remain inherently nonspecific and may represent generalized systemic or age-related alterations rather than direct indicators of central nervous system pathology [38,39]. Therefore, interpretation of tear-based inflammatory or oxidative markers should be undertaken cautiously, particularly in aging veterinary populations where comorbidities are common.

#### 5.3.2. Potential Detection of CNS-Specific Proteins in Tear Film

Validated CNS-specific biomarkers such as neurofilament light chain (NfL) and glial fibrillary acidic protein (GFAP) are detectable in peripheral blood and reflect neuroaxonal injury and astroglial activation in human neurodegenerative diseases [38,39,45,46]. Given their measurable presence in systemic circulation, investigation of these markers in tear fluid represents a plausible translational direction.

The lacrimal system is highly vascularized and immunologically active, providing potential pathways for the transfer of low-molecular-weight proteins from systemic circulation. Moreover, the neuroanatomical proximity of ocular structures to the central nervous system supports the hypothesis that tear fluid may reflect neurodegenerative processes beyond systemic inflammation.

Although direct evidence of validated CNS-specific biomarkers in canine tear fluid remains limited, plasma NfL concentrations have been shown to increase in aging dogs and in canine cognitive dysfunction (CCD) [41,42]. In addition, amyloid-β peptides have been detected in plasma and CSF of aged dogs with cognitive impairment [49,50], further supporting translational parallels between Alzheimer’s disease and CCD.

Proteomic analyses of tear fluid have identified proteins involved in cytoskeletal organization, proteostasis, and immune regulation [53], pathways mechanistically aligned with axonal injury, synaptic dysfunction, and glial activation described in neurodegeneration [43,47,48].

#### 5.3.3. Translational Perspective: From Alzheimer’s Disease to Canine Cognitive Dysfunction

Human studies have reported altered tear protein profiles in patients with Alzheimer’s disease, including changes in proteins associated with amyloid processing, inflammation, and cellular stress [53]. Given the recognized neuropathological similarities between Alzheimer’s disease and canine cognitive dysfunction, including amyloid deposition and age-related neurodegeneration [44,50], tear-based biomarker research in dogs represents a promising translational model.

Importantly, integration of tear proteomics with validated blood and CSF biomarkers—such as NfL [41,42] or amyloid-β peptides [49,50]—may improve disease specificity and facilitate multimodal biomarker panels. Such combined approaches may enhance early detection, monitoring of progression, and therapeutic evaluation in veterinary neurodegeneration.

#### 5.3.4. Current Limitations and Future Directions

Despite its promise, tear biomarker research in veterinary neurology remains in an exploratory phase. Standardization of tear collection protocols, age-related variability, systemic confounders, and limited longitudinal validation studies represent significant challenges.

Future research should prioritize targeted evaluation of validated CNS-specific biomarkers—particularly markers of axonal injury (NfL) [38,39,41,42], astrocytic activation (GFAP) [45,46], synaptic degeneration [43], and lysosomal/autophagic dysfunction [48]—within tear fluid matrices. Longitudinal studies integrating cognitive scoring, imaging, and multimodal fluid biomarkers will be essential to establish clinical utility.

By moving beyond broad inflammatory or oxidative categories toward mechanistically informative CNS-specific targets, tear film proteomics may evolve into a clinically meaningful tool in veterinary neurodegenerative research.

### 5.4. Clinical Applications and Diagnostic Potential

If validated, tear-derived biomarkers could offer several important advantages in the clinical management of cognitive dysfunction. Tear sampling may facilitate early screening of geriatric patients, support differentiation between cognitive dysfunction and other neurological or systemic disorders, and allow objective monitoring of disease progression over time. In addition, tear proteomics may prove valuable for assessing responses to dietary, pharmacological, or behavioral interventions aimed at slowing cognitive decline.

Importantly, the integration of tear biomarkers with standardized behavioral assessment tools and clinical scoring systems may improve diagnostic accuracy and provide a more comprehensive evaluation of cognitive status in companion animals. Such multimodal approaches are increasingly recognized as important for the effective management of chronic neurodegenerative disorders.

## 6. Methodological Limitations and Future Research Directions

Despite the growing interest in tear film proteomics as a source of biomarkers for cognitive dysfunction and neurodegenerative disorders in companion animals, several methodological and conceptual limitations currently restrict its clinical translation.

A major challenge arises from the intrinsic biological variability of tear film composition. Tear protein profiles are influenced by numerous pre-analytical factors, including collection technique, sampling duration, time of day, environmental conditions, ocular surface integrity, and recent ophthalmic manipulation. In addition, age, breed, sex, and individual physiological variability substantially affect tear proteomes, complicating the identification of disease-specific signatures in geriatric populations.

Another important limitation concerns the lack of standardized protocols for tear collection, processing, and proteomic analysis in veterinary medicine. Differences in collection devices (e.g., Schirmer strips versus microcapillary tubes), elution buffers, protein extraction methods, enzymatic digestion procedures, mass spectrometric platforms, and bioinformatic pipelines reduce inter-study comparability and may lead to inconsistent protein identification. The small sample volume typically obtained from dogs and cats further constrains analytical sensitivity and may bias detection toward highly abundant proteins, potentially obscuring low-abundance biomarkers of greater mechanistic relevance.

Interpretative challenges also arise from incomplete annotation of canine and feline proteomes and limited availability of species-specific protein databases. These constraints may affect accurate protein identification and functional pathway analysis, particularly when investigating CNS-specific biomarkers or subtle quantitative differences associated with cognitive decline.

From a clinical perspective, the current evidence base remains limited. Most tear proteomic data derive from healthy animals or from studies focused on ocular surface disorders rather than naturally occurring cognitive dysfunction. Direct comparisons between cognitively normal and cognitively impaired dogs and cats are scarce, and longitudinal studies assessing temporal changes in tear proteomes during disease progression are largely lacking. Consequently, causal relationships between tear-derived proteins and central neurodegenerative processes remain speculative.

Furthermore, geriatric patients frequently present with concurrent systemic diseases, medications, and age-related comorbidities that may influence tear composition and act as confounding variables. Careful phenotyping, rigorous exclusion criteria, and appropriate control groups will therefore be essential in future biomarker validation studies.

Only through systematic methodological refinement, cross-validation across biological matrices, and robust clinical correlation can tear film proteomics evolve from an exploratory research tool to a clinically meaningful diagnostic and monitoring approach in veterinary neurodegenerative medicine.

## 7. Conclusions

Cognitive dysfunction and neurodegenerative disorders represent an increasing clinical challenge in aging dogs and cats, yet objective and minimally invasive biomarkers suitable for early detection and disease monitoring remain limited. Tear film proteomics has emerged as a promising and accessible approach for exploring peripheral molecular alterations associated with neurodegeneration in companion animals.

While early studies primarily identified tear-derived proteins involved in inflammation and oxidative stress, recent advances in neurodegenerative biomarker research emphasize the importance of CNS-specific and mechanistically informative markers. Biomarkers reflecting axonal injury (e.g., neurofilament light chain), synaptic degeneration, astrocytic activation (GFAP), microglial activation, and proteostatic dysfunction provide a more precise framework for interpreting peripheral proteomic alterations in the context of central nervous system pathology.

Current evidence in veterinary medicine remains largely exploratory; however, translational data from canine cognitive dysfunction and human Alzheimer’s disease suggest that integration of tear proteomics with validated blood and cerebrospinal fluid biomarkers may enhance specificity and biological relevance. Rather than relying solely on broad and nonspecific categories such as oxidative stress or general inflammation, future research should prioritize targeted evaluation of CNS-specific biomarkers within tear fluid matrices.

Progress in this field will depend on standardized methodologies, well-characterized longitudinal cohorts, and multimodal integration of behavioral, imaging, and molecular data. With rigorous validation and cross-matrix correlation, tear film proteomics may evolve from an exploratory research tool into a clinically meaningful component of diagnostic and monitoring strategies for neurodegenerative disorders in dogs and cats.

## Figures and Tables

**Table 1:** Major tear-derived protein categories associated with biological processes implicated in neurodegeneration in dogs and cats.

Biological Process	Representative Tear Proteins Identified in Dogs and Cats	Relevance to Neurodegeneration
Neuroinflammation	Complement components (e.g., C3, C4); S100 proteins; immunoglobulins	May reflect systemic or neuroimmune activation accompanying cognitive decline
Oxidative stress	Peroxiredoxins; superoxide dismutase; glutathione-related enzymes	Indicate redox imbalance associated with aging and increased neuronal vulnerability
Proteostasis and cellular stress response	Heat shock proteins; ubiquitin-related proteins	Suggest alterations in protein folding and degradation pathways implicated in neurodegenerative disorders
Cytoskeletal organization	Actin-binding and structural proteins	May indirectly relate to neuronal structural alterations observed in cognitive dysfunction
Immune regulation	Lysozyme; lactoferrin; acute-phase proteins	Reflect systemic immune modulation potentially accompanying neurodegenerative processes

**Table 2:** CNS-Specific Biomarkers of Neurodegeneration Relevant to Veterinary Medicine and Their Potential Applicability to Tear Film Proteomics.

Pathophysiological Process	Biomarker	Matrix (Human)	Evidence in Dogs	Validation Status	Key References
Axonal injury	NfL	CSF, Plasma	Reported in aging dogs and CCD	Validated (human), emerging (dog)	[38,39,41,42]
Axonal injury	pNfH	CSF	Limited	Exploratory	[38]
Synaptic degeneration	neurogranin	CSF	Not validated	Validated (human)	[43]
Synaptic degeneration	SNAP-25	CSF	Not validated	Validated (human)	[43]
Astrocyte activation	GFAP	CSF, Plasma	Limited veterinary data	Validated (human)	[45,46]
Microglial activation	sTREM2	CSF	Not validated	Validated (human)	[47]
Microglial activation	YKL-40	CSF	Not validated	Validated (human)	[51]
Amyloid pathology	Aβ42/Aβ40	CSF, Plasma	Reported in CCD	Established (human), emerging (dog)	[49,50,52]
Proteostasis dysfunction	Cathepsins	CSF	Theoretical	Emerging	[48]

## Data Availability

No new data were created or analyzed in this study. Data sharing is not applicable to this article.
